# Comparison of Performance Properties and Prediction of Regular and Gamma-Irradiated Granular Waste Polyethylene Terephthalate Modified Asphalt Mixtures

**DOI:** 10.3390/polym13162610

**Published:** 2021-08-06

**Authors:** Aliyu Usman, Muslich Hartadi Sutanto, Madzlan Napiah, Salah E. Zoorob, Nura Shehu Aliyu Yaro, Muhammad Imran Khan

**Affiliations:** 1 Department of Civil and Environmental Engineering, Universiti Teknologi PETRONAS, Bandar Seri Iskandar, Perak 32610, Malaysia; muslich.sutanto@utp.edu.my (M.H.S.); madzlan_napiah@utp.edu.my (M.N.); nura_19001733@utp.edu.my (N.S.A.Y.); muhammad_17007177@utp.edu.my (M.I.K.); 2 Construction and Building Materials Program, Kuwait Institute for Scientific Research, P.O. Box 24885, Safat 13109, Kuwait; szoorob@kisr.edu.kw

**Keywords:** irradiated waste polyethylene terephthalate, stiffness, rutting, ANN-RSM, gamma-irradiation

## Abstract

The utilization of waste polyethylene terephthalate (WPET) as aggregate substitutes in pavement has been extensively promoted because of its environmental advantages. However, previous studies have shown that a high percentage of WPET reduces the performance of the pavement. To increase the durability of pavement and mitigate the environmental issues caused by WPET, WPET is treated with gamma-irradiation as a component in asphalt mixtures. The study objectives were to investigate the feasibility of using WPET granules as a sustainable aggregate on asphalt mixture stiffness and rutting and predict the asphalt mixture performance containing irradiated WPET via an RSM-ANN-framework. To achieve the objectives, stiffness and rutting tests were conducted to evaluate the WPET modified mixtures’ performance. The result indicated that samples containing 40% irradiated WPET provided a better performance compared to mixtures containing 20% non-irradiated WPET, increasing the stiffness by 27% and 21% at 25 °C and 40 °C, respectively, and rutting resistance by 11% at 45 °C. Furthermore, both predictive models developed demonstrated excellent reliability. The ANN exhibited superior performance than the RSM. The utilization of WPET as aggregate in asphalt mixtures represents a way to addressing related recycling issues while also improving performance. With gamma-irradiation treatment, the utilization of WPET can be increased with improved asphalt mixture performance.

## 1. Introduction

The growing waste material production on the globe each day is one of the most critical environmental concerns. Globally, plastic waste is considered a serious challenge. An Australian Plastics Recycling Study, for example, reported the overall consumption of plastics to be approximately 3.5 million tons in 2016–2017, although only 415,200 tons were recycled [1,2]. The design of road pavements also requires the exploration of high quantities of virgin materials (mostly aggregates) and mass transport, resulting in increased greenhouse gas emissions [3,4]. The demand for material diversification in road pavements, especially in developing countries, can also be attributed to the growing need for improved efficiency, longevity, and minimize costs of rehabilitation and construction [3]. The significant population growth leads to an increase in vehicle traffic and axle loads, leading to road section premature defects such as rutting and fatigue cracking [3,5,6,7].

Currently, experts are increasingly involved in repurposing waste materials as alternatives to non-renewable conventional materials in the construction of green and sustainable pavements as a result of environmental awareness of the conservation of natural resources between the rising needs of natural aggregates from the highway industry and the environmental and health-related problems arising from the large volume of waste generation worldwide. One of the recent wastes of interest is polyethylene terephthalate (PET) due to its non-biodegradability nature and other favorable physical and chemical properties.

Polyethylene terephthalate (PET) is a type of thermoplastic polymer that possesses both crystalline and amorphous portions (semi-crystalline) [8] with high mechanical strength, hydrolytic, thermal, and chemical resistance. It is being utilized in the manufacturing process [9] as a precision molding for office and household items, automobile components, and electronic devices. The need is continually rising because of the convenience of PET bottle usage. Approximately 99% of plastics are manufactured from chemicals extracted from coal, oils, and non-renewable natural gas. Therefore, it is paramount to devise a means of managing these vast quantities of PET waste in an eco-friendly manner. One such course is by incorporating the waste PET in highway and cement concrete industries as secondary materials.

Although waste PET has been studied by various researchers as aggregates replacement in cement concrete production [9,10,11,12,13,14,15], limited studies concentrated on the utilization of waste PET as aggregates in asphalt pavement production with mixed outcomes [16,17,18,19]. It is well-known that ionizing radiation such as gamma rays contributes to polymer chain cross-linking as well as scission. The crystalline structure is also altered after the polymer exposure to gamma irradiation. The increase in the degree of crystallinity of polymers that are exposed to gamma rays resulted in making the polymer tougher, stiffer, and harder when compared to non-irradiated ones [13].

Moghaddam, Soltani [20] explored a statistical method to investigate the influence of various parameters such as applied stress levels, the temperature of testing, and PET content on the stiffness characteristics of asphalt mixture. The investigation highlighted that the asphalt mixture’s stiffness was influenced by the magnitude of stress applied and the content of PET. However, the stiffness of the mixture was more prone to temperature fluctuations. The result also showed that at lower temperatures, the PET modification had a pronounced impact on the asphalt mixture’s stiffness. Furthermore, the result showed that the PET variation was more significant on the stiffness relative to the stress levels. It was concluded that 0.41% PET was the optimum for SMA mixtures to achieve optimal stiffness. Additionally, several investigations have been carried out to determine the impact of PET on asphalt pavement rutting resistance. The outcomes of these research works showed that rutting behavior could be enhanced by incorporating PET [21,22,23]. In this context, Ziari, Kaliji [23] suggested that, by adding PET content, the rutting resistance of mixes improved, and with an increase in the particle size of the PET, the rutting resistance reduced. It can be concluded that the mechanical performance characteristics of asphalt concrete blends could be enhanced by using a low PET amount. Additionally, the mechanical performance characteristics of asphaltic pavements could be adversely affected by adding the PET quantity above the optimal concentration [22,23,24,25].

In the field of asphalt concrete materials, response surface methodology (RSM) and artificial neural network (ANN) methods have recently been used to predict or model their performance [26,27,28,29,30,31,32,33,34]. However, only a few studies have employed it to repurpose irradiated waste polyethylene terephthalate (WPET) in asphaltic concrete [35,36,37]. RSM is a statistical technique commonly used to design experiments, modeling, and operation optimization by measuring the individual and the interaction effects of various factors (autonomous parameters) in a lesser range of experiment iterations [37,38]. An artificial neural network (ANNs), on the other hand, is a structure of a black box popularized by manner biological neuron functions. ANN models utilize specialized training algorithms in an additional data set to understand the pattern and implement an activation function to predict targeted outcomes via an iterative learning method using parameter values [39,40]. On this premise, ANN, a nonlinear, nonparametric modeling approach that provides a high level of precision to solve complex problems of nonlinear relationships, has gained the interest of material science and pavement engineering experts.

Therefore, the main objective of the present work was to partially substitute the natural aggregate with regular waste PET (RWPET) and irradiated waste PET (IWPET) by volumetric substitution in densely graded asphaltic concrete (AC) mixes for comparison and evaluating the impact of gamma-irradiation treatment on the waste plastic on the stiffness and rutting characteristics. In an attempt to improve the waste PET mechanical and thermal characteristics, the waste PET granules were treated with gamma-irradiation, an efficient and creative approach to recycling in pavement construction. Microstructural characterization tests utilizing X-ray diffraction (XRD), differential scanning calorimetry (DSC), and Fourier transformed infrared spectroscope (FT–IR) were also implemented on the RWPET and IWPET particles to examine the influence of gamma-irradiation treatment. Furthermore, RSM and ANN statistical tools were employed to optimize and estimate the mechanical performance properties of asphalt concrete mixture containing IWPET granules as substitutes for natural aggregates. A comparison was made based on the RSM and ANN with a structure optimized by RSM models given predictability and reasonable accuracy, considering the statistical performance index parameters of both models such as coefficient of the determination (R^2^), root mean square error (RMSE), and mean relative error (MRE). The uniqueness of the present study is the comparison of the two optimization techniques to predict the best outcomes with best-operating variables for IWPET-modified asphalt concrete blends with fewer test run numbers, which could save time and reduce the experimental tests.

## 2. Materials and Methods

### 2.1. Materials

#### 2.1.1. Aggregate and Asphalt Binder

Crushed granite aggregates obtained from Sunway quarry, located in Kampar, Perak Malaysia, were used for producing the asphalt concrete blends. The physical properties of the conventional aggregates are summarized in Table 1. The asphalt binder utilized in this investigation was a penetration grade 60/70 supplied by PETRONAS refinery, Melaka, with its physicochemical characteristics also summarized in Table 1.

#### 2.1.2. Waste Polyethylene Terephthalate (WPET)

Waste polyethylene terephthalate (WPET) granules were procured from the Enhanced plastic industry; Perak was used to substitute a portion of conventional mineral aggregates partially by the volume of equal size in densely graded asphalt concrete mixes. The WPET had a high melting point of around 250 °C and a glass transition temperature of 92.8 °C. The specific gravity and water absorption of the WPET were also 1.35 g/cm^3^ and 0.11%, respectively. The WPET particle size distribution is presented in Table 2.

### 2.2. Gamma-Irradiation of WPET

Non-plastic and other impurities associated with recycling facility imperfections were sorted out manually. A ^60^Cobalt gamma irradiator that operates at 58 Gy/min located at the Sinagama Nuklear Agensi, Malaysia, was used to irradiate the sorted WPET. A 100 kGy gamma radiation dosage was selected for this study based on preliminary tests and the findings of previous investigations [13,37,41,42,43].

### 2.3. Microstructural Characterizations

X-ray diffraction (XRD) analysis was carried out to identify and measure the amorphous and crystalline material phases. XRD analysis was performed utilizing an advanced Bruker D8 X-ray diffractometer (Bruker, Billerica, MA, USA) that worked at 40 kV and a 40 mA current supply utilizing Ni-filtered Cu Kα radiation with a graphite monochromator (λ = 1.5406 Å) at a 2θ angle of 5–80°. To identify the functional group as well as the molecular fingerprints of the materials before and after gamma-irradiation, Fourier transformed infrared spectroscope (FT–IR) was performed on the RWPET and IWPET. With a resolution of 2 cm^−1^ and 40 scans, using the Perkin Elmer FT–IR spectrometer (Perkin Elmer Spectrum 65, MA, USA), the FT–IR spectra were recorded in the wavenumber range of 4000 cm^−1^ to 500 cm^−1^ at a scanning rate of 64/min, measurements were taken. In order to analyze the thermal behavior of the WPET before and after gamma-irradiation, a thermogravimetric analysis (TGA) and differential scanning calorimetry (DSC) were carried out, respectively, using the TGA, Model SHIMADZU DTG−60/60H, Kyoto, Japan and DSC instruments (Model DSC Q2000 V24.11, Oberkochen, Germany).

### 2.4. Sample Preparations

Initially, the Marshall mix design approach was employed to evaluate the optimum asphalt content (OAC) and the asphalt mixtures’ volumetric properties for the reference and modified asphalt concrete mixtures incorporating 10%, 20%, 30%, and 40%, respectively, of RWPET and IWPET granules by volume of equal size according to AASHTO T245 [44]. Nine (9) different types of mixes, including the reference mixture and four each for the RWPET and IWPET, modified asphalt blends were designed for analysis and comparison. In this study, the amounts of both WPET granules were incorporated as a substitute for the virgin mineral aggregate having different volumetric contents. The idea behind this substitution was to replace a portion (by volume) of the conventional fine aggregate with an equal amount of RWPET and IWPET from the original aggregate particle size distribution and based on the lower specific gravity of the waste material in comparison to the conventional mineral aggregate, which was aimed at having consistent mixes. All prepared mixes were made to achieve 3–5% air void content. To make the modified mixes, the modified dry process was utilized. The binder and aggregates were mixed until the aggregate particles were well coated, after which the waste plastic granules were added and further mixed until the added waste plastic granules were fully coated with the asphalt binder. Maintaining the waste plastic granules in the mixture in their natural state (semi-crystalline resin) with minor changes in their shapes and properties was the main reason for choosing this method. According to the Marshall mix design method, the volumetric and Marshall characteristics including bulk specific density (BSD), air void (AV), voids in mineral aggregate (VMA), voids filled with asphalt (VFA), Marshall stability (MS), and Marshall flow (MF) were obtained for different asphalt content (AC) by weight of total mix and presented in Table 3. The wheel tracking slab specimens with dimensions 305 × 305 × 50 mm and cylindrical Marshall specimens, respectively, for the rutting and stiffness modulus determination were prepared at the obtained OAC for each mixture type.

### 2.5. Wheel Tracking and Stiffness Modulus Tests

A simulative test to predict the rutting in an asphalt concrete mixture subjected to repeated loading is the wheel tracking test. The test was used to determine the rutting characteristics of both the reference and modified asphalt mixes prepared with RWPET and IWPET granules as substitutes for natural aggregates. Slab-shaped specimens were prepared for the wheel track test, and each slab-shaped specimen was prepared by mixing approximately 10 kg of aggregates (coarse, fine, and mineral filler) for reference mixture at OAC. On the other hand, for the modified asphalt mixtures, the required volume of aggregates, and the waste plastic granules were mixed at OAC. In this research work, the Wessex wheel tracking device (Model S867, Wessex Test Equipment, Banwell, Weston-super-Mare, North Somerset, UK) was utilized to evaluate the resistance to rutting of the mixes following BS 598-110 [45]. For this study, 87 slabs (27 slabs for comparison of the effect of exposing WPET granules to gamma-irradiation with non-irradiated ones and 60 slabs for statistical modeling) were prepared at a mixing temperature of 155 ± 5 °C, three for each mixture type using an electric asphalt concrete mixer and compacted to achieve air void content of 7 ± 2% according to BS 598-110 with a kneading compactor. While the stiffness modulus test is used to evaluate the quality of material and investigate the effect of temperature and load on durability and flexibility. The outcome of this test was a significant input in mechanistic pavement design, evaluation, and analysis [46]. In this study, the quality of the Marshall-compacted samples produced with and without WPET were evaluated following the BS EN 12697-26(E) [47] procedure. Samples were pre-conditioned at the testing temperature for 4 h before centrally positioned on the test jig. The loading plate was properly fitted on top of the sample to ensure the symmetrical distribution of the imposed load. The height and diameter of the samples obtained at six different positions were inserted into the computer system attached to the Universal Material Testing Apparatus (MATTA), IPC Global UTM-30 Servo−Hydraulic Universal Testing Machine (Model 79−PV70B12/I2), Control Group, Milan, Italy.

### 2.6. Statistical Models

#### 2.6.1. Response Surface Methods

RSM is a set of mathematical and statistical techniques that measures the interactions among various input variables and one or more dependent factors (responses) while optimizing the conditions of the relevant parameters and predict the best response [48]. In this study, Stat-Ease Inc. developed software; Design Expert (V11) was utilized for the experimental design, statistical analysis, mathematical modeling, and optimization. The central composite design (CCD) approach was used for the design of the experiment (DoE) based on two independent factors (temperature and IWPET content). Central Composite Design (CCD) is the most widely used and efficient method used to statistically evaluate the interaction among the independent variables and the responses within the experimental range [31,38,49,50,51]. For the dependent variable stiffness modulus, the independent variable test temperature was varied in three levels (10 °C, 25 °C, and 40 °C), and IWPET contents were varied in five levels (0%, 10%, 20%, 30%, and 40% replacement by volume of equal size), as illustrated in Table 3. While for the dependent parameter rutting, the independent parameters test temperature was also varied in three levels (40 °C, 50 °C, and 60 °C), and the same levels were utilized as that of the stiffness for IWPET amount, as presented in Table 4. The levels and ranges of the independent factors utilized were chosen based on the standard requirements and selected works of previous studies [16,17,19,20,45,47,52,53,54].

Twenty (20) mixtures with nine duplications at the central point were developed based on the RSM DoE. The numbers of duplication points were evaluated by the software which was used for pure error calculation.

Using the asphalt Universal Testing Machine (UTM-30), the stiffness modulus test was conducted, and the results were entered in the RSM for design. Similarly, the Wessex wheel tracking machine was utilized to evaluate the rutting, and the experimental outcomes were inputted into the RSM for analysis. The prediction of optimal independent factors was achieved after performing CCD analysis using a suitable polynomial model with terms as shown in Equation (1).

(1) $$Y=b_{o}+\overset{n}{\underset{i=1}{\sum }}b_{i}X_{i}+\overset{n}{\underset{i=1}{\sum }}b_{ii}X_{i}^{2}+\overset{n}{\underset{i=1}{\sum }}\overset{n}{\underset{j=1}{\sum }}b_{ij}X_{i}X_{j}+\varepsilon$$

where *Y* is the response (Stiffness and rut depth); *b_0_* is the fixed experiment response value of the central point; *X_i_* and *X_j_* represent independent variables (test temperature and percentages of IWPET); the linear and quadratic terms are represented by coefficients *b_i_* and *b_ii_*. The interaction term coefficient is denoted as *b_ij_,* and the random error of the model and the studied numbers of the factors are denoted as *ε* and *n*, respectively.

The model terms were selected or rejected based on the probability (P) value having a 95% confidence level, and variance analysis (ANOVA) was used to determine the outcomes. The regression coefficient (R^2^) was used to assess the consistency of the selected model’s fit. Using Fisher’s F-test, the statistical significance of the fitted model selected was tested. Based on the effects of the independent parameters, the 2D contour and 3D plots of the response variables were obtained.

Verification was then carried out to evaluate the response outputs obtained statistically and validate the equations gotten from RSM. The verification was carried out using approximate values from numerical RSM optimization by conducting an added experiment. Through numerical optimization, the optimal conditions were achieved, which can be used for the RSM design outcomes validation.

#### 2.6.2. Artificial Neural Network

The basic elements of the ANNs are known as ‘‘neurons’’, which are the processing elements of ANNs and consist of many simple computational elements that are connected [26,55,56]. The “network” is defined as the structure in which the neurons act simultaneously in a group [26,57]. Different ways have been developed to enhance the estimation precision of the ANN model; the backpropagation algorithm is one of the most preferred methods in this category [55,58]. The network includes an input layer, one or more hidden layers, and the output layer. Each artificial neuron receives and processes information entering from other neurons and then relays the signals to other neurons. In this analysis, the same 20 runs of data sets identified by RSM were also employed for applying the neural network methodology. A total of 60% of the data (i.e., 12 runs) were used for training the neural network, 20% (4 runs) each for the cross-validation and testing the neuron, respectively. The backpropagation learning algorithm was used in feed-forward with one hidden layer. The logarithmic sigmoid transfer function was used as the activation function for hidden layers and output layers. The data splitting into training, cross-validation, and testing sets was performed to prevent overtraining and over-parameterization. By modifying the neuron’s weight to achieve the best fit in terms of determination coefficient (R^2^) and root mean square error (RMSE) values, the network was trained to learn the data pattern. The two models’ superiority was evaluated via three statistical performance index parameters: roots mean square error (RMSE), mean relative error (MRE), and coefficient of determination (R^2^), which were determined by utilizing Equations (2)–(4), respectively:

(2) $$RMSE=\sqrt{\frac{1}{n}\overset{n}{\underset{i=1}{\sum }}{\left(T_{i}-O_{i}\right)}^{2}}$$

(3) $$MRE=\frac{1}{n}\overset{n}{\underset{i=1}{\sum }}\left|100\frac{T_{i}-O_{i}}{T_{i}}\right|$$

(4) $$R^{2}=1-\left(\frac{\sum_{i=1}^{n}{\left(T_{i}-O_{i}\right)}^{2}}{\sum_{i=1}^{n}{\left(O_{i}\right)}^{2}}\right)\;$$

where *n* is the number of points in the data group; *T* is the actual response; *O* is the predicted response value.

## 3. Results and Discussions

### 3.1. Morphological Characterizations

**Figure 1:**
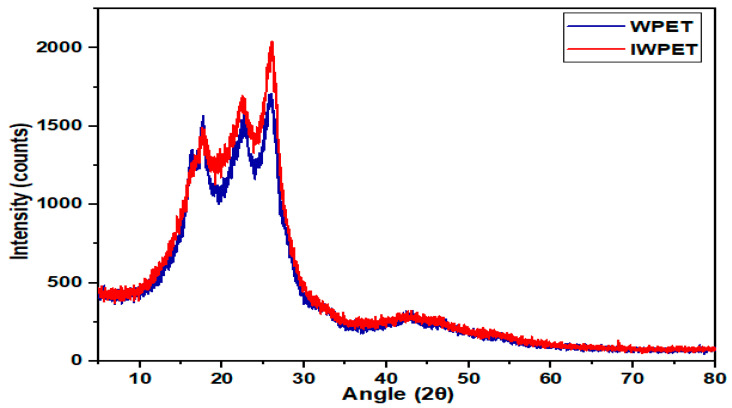
X-ray Diffraction (XRD) diffraction plot for regular waste PET (RWPET) and irradiated waste PET (IWPET).

The WPET particles were subjected to powder X-ray Diffraction (XRD) before and after exposure to gamma irradiation to measure the crystalline degree and the corresponding amorphous composition of the waste plastics material. To characterize amorphous and crystalline degrees in materials, XRD is the most appropriate and efficient analytical instrument. WPET before and after exposure to irradiation were analyzed to evaluate the impact of irradiation on crystallinity and amorphous percentages of plastics. The crystallinity and amorphous percentages of the RWPET and IWPET are depicted in Figure 1. As depicted in the diffractogram, the IWPET had larger intensity peaks compared to the RWPET indicating a larger area under the curve and hence, higher crystallinity. The crystalline percentage of the plastic material was calculated by dividing the area of crystalline peaks by the total area under the plot. The analysis revealed that WPET crystallinity rose from 41.15% to 46.07% upon gamma irradiation.

**Figure 2:**
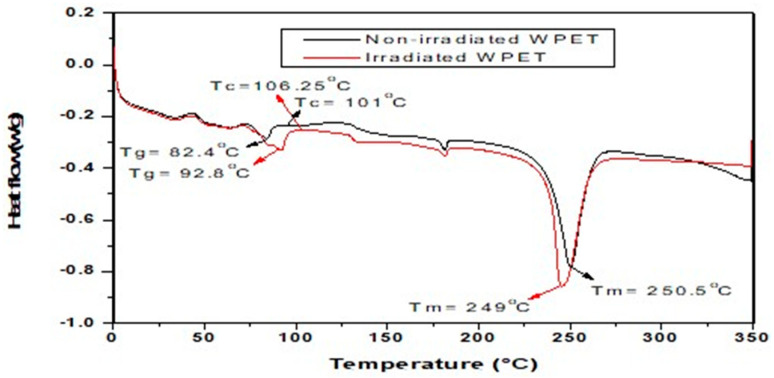
Differential scanning calorimetry (DSC) thermogram curve for waste polyethylene terephthalate (WPET) before and after gamma irradiation.

Figure 2 shows the DSC curve of the material before and after treatment with gamma irradiation. As can be seen from the curve, when heated at room temperature, these two materials underwent three phases, including glass transition temperature (T_g_) about 82.4 °C and 92.8 °C; the cold crystallization temperature (T_c_) at 101 °C and 106.25 °C; and the melting point temperature (T_m_) at 250.5 °C and 249 °C, respectively, for the materials before and after exposure to gamma irradiation. At the T_g_, the substance underwent a structural adjustment from a glassy rigid state to a rubbery, flexible form of much greater molecular mobility. The large-scale molecular motion was not possible below the T_g_, since at that point the substance was frozen. As the temperature increased above the T_g_, there were segmental motions in the polymer chain, which caused the material to be soft or rubbery. The material’s crystal growth then followed at the temperature of cold crystallization, and subsequently, crystal melting at the sample melting point. This behavior of increased T_g_ value might be due to the increase in the intensity of the diffraction peak, which resulted in an increase in the degree of crystallinity after gamma irradiation exposure and thus, reduced the overall degree of enthalpic relaxation. As the material was gamma-irradiated, the T_c_ value increased, which might result in a higher heat flux producing a highly arranged crystalline structure and a higher amount of heat to be melted (phase transition). After exposure to gamma rays, the material became more stable thermally compared to the non-exposed material, with anticipated amorphous enhancement and ultimate strength properties.

**Figure 3:**
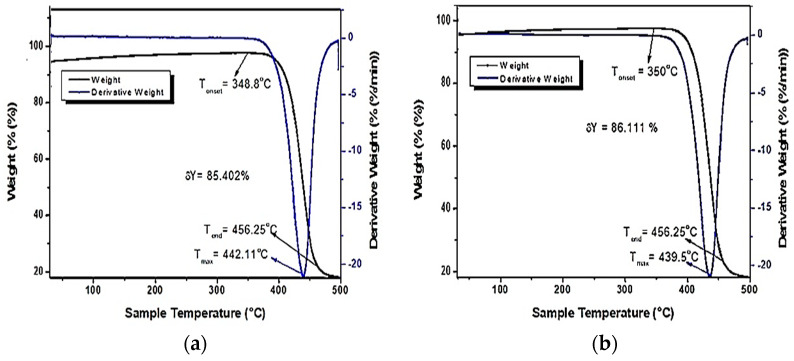
TG/DTG thermograms for WPET (**a**) before gamma−irradiation (**b**) after gamma−irradiation.

Utilizing the TGA instrument, the thermal stability of the RWPET and IWPET was evaluated. Figure 3 displays the TG/DTG thermograms of the non-irradiated and irradiated plastic material. The plastic material demonstrated one degradation level, as can be seen from the thermogram, with initiation and peak temperatures of about 350 °C and 439.5 °C for IWPET and 348.8 °C and 442.11 °C for RWPET. The effect of crosslinking that occurred on the material after the gamma-irradiation treatment caused the material onset temperature (Tonset) to increase from 348.8 °C for RWPET to 350 °C for the IWPET; thus, IWPET granules having higher onset temperature (Tonset) had higher heat to disintegrate, while RWPET granules compared to the IWPET granules appeared to breakdown more quickly when exposed to heat [9]. This behavior suggested that the RWPET’s initial thermal decomposition was marginally lower than the IWPET’s. Based on the materials’ initiation temperature (T_onset_), it could be concluded that after irradiation treatment, the WPET was thermally more stable, which happened as a result of ionizing radiation caused by the crosslinking effect.

**Figure 4:**
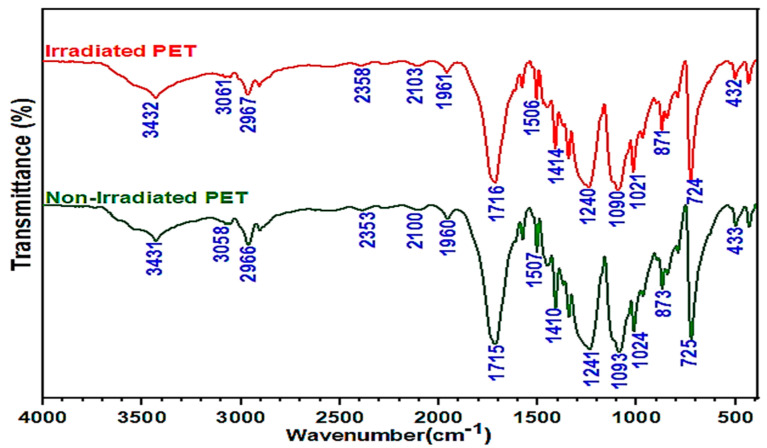
Fourier transformed infrared spectroscope (FT−IR) spectrum of regular and irradiated WPET.

The chemical structural analysis of the RWPET and IWPET granules was evaluated via the utilization of FT−IR spectroscopy. As shown in Figure 4, the WPET and IWPET sample patterns showed similar transmittance bands, showing that irradiation treatment did not substantially affect the WPET chemical bonds. The description of some extreme peaks’ bands identifying relevant functional groups was presented. The intense spectrum bands at wavenumber 1715.36 cm^−1^ indicated C=O stretching from the carboxylic acid group, at 1239.93 cm^−1^ the terephthalate group was represented. The wavenumber 1089.68 cm^−1^ was attributed to the polyethylene methylene group, and benzene ring and polar ester group interactions were represented at 724.18 cm^−1^. Other minor spectra were hydroxyl group and CH symmetrical stretch represented by wavenumber 3431.73 cm^−1^ and 2965.52 cm^−1^, respectively.

### 3.2. Stiffness and Rutting of Asphalt Mixtures Containing RWPET and IWPET Granules

#### 3.2.1. Stiffness Modulus of RWPET and IWPET Modified Asphalt Mixtures

**Figure 5:**
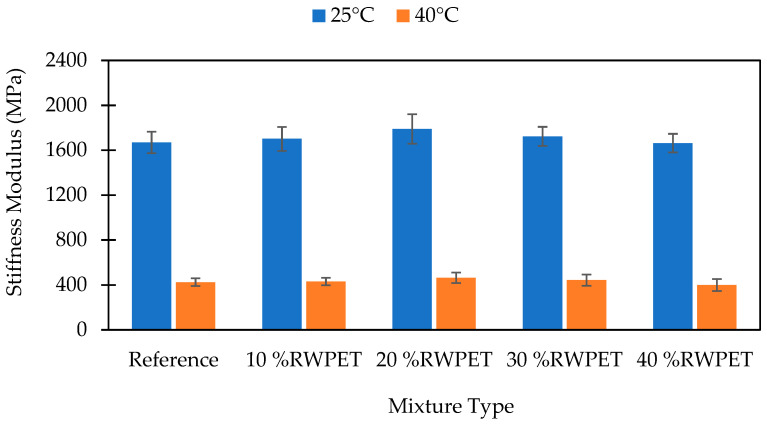
Effect of RWPET on stiffness modulus at 25 °C and 40 °C.

**Figure 6:**
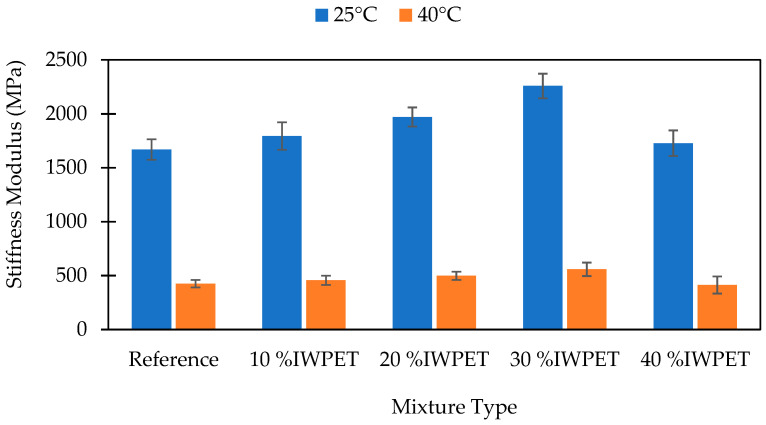
Effect of IWPET on stiffness modulus at 25 °C and 40 °C.

The stiffness modulus of the reference and WPET modified asphalt mixes are illustrated in Figure 5 and Figure 6. It can be noted that both the RWPET and IWPET modified asphalt mixes’ stiffness modulus tested at 25 °C increased as the WPET content increased up to an optimal dosage of 20% and 30%, respectively, after which the stiffness values were observed to recess with an additional increase in the concentration of the WPET granules as a substitute for natural aggregate. Additionally, the granules content was high at 40% for both modified asphalt mixes resulting in the loss of internal friction attributed to the lower friction surface of the WPET particles. This finding coincided with the research reported by [38]. It is noticeable from the results obtained that the stiffness of the RWPET and IWPET modified asphalt concrete mixes were higher than the reference mix. Similarly, the same pattern was noted when the stiffness was tested at an elevated temperature of 40 °C.

The increase in the stiffness modulus values for the RWPET and IWPET modified asphalt concrete mixes indicated an enhancement in the elastic property with an increase in the amount of the WPET granules. This improvement in elastic property may probably be due to the semi-crystalline nature of the material utilized and possibly the adopted method of adding the WPET into the asphalt mix. The amorphous portion of the material softened at its glass transition temperature of 82.4 °C and 92.8 °C for RWPET and IWPET particles, respectively, which mixed with the asphalt binder and increased its cohesion. On the other hand, the crystalline part remained intact during blending for both RWPET and IWPET particles and acted as elastic aggregates, which result in a stiffer asphalt mixture with increased stiffness. For the IWPET particles as seen in the XRD diagram, there was an increase in the crystalline degree because of the influence of exposing the WPET to gamma-irradiation. The decrease in the stiffness modulus values of both modified asphalt mixtures at 40% WPET contents could be because of the lower rigidity of the material compared to the natural aggregate replaced and its mechanical properties in the mix [20]. Consequently, the modified asphalt mixes became more flexible and hence, yielded higher tolerance to deformation under load applications [25,59].

As shown in Figure 5 and Figure 6, the stiffness modulus values of the asphalt samples decreased as the testing temperature was increased from 25 °C to 40 °C. The reduction in the stiffness values with increasing test temperature occurred due to the change in the asphalt binder viscosity resulting from the temperature increase, which caused particle slippage in asphalt concrete mixes. This decreasing stiffness behavior was obtained for both RWPET and IWPET modified asphalt mixes. However, compared to the reference mix, the stiffness modulus values of the modified asphalt samples were higher as the temperature increased with the presence of the WPET granules in the mixtures, which could resist the particle slippage because of the increased temperature. The resistance towards particle slippage resulted in reducing the rate of decrease in the stiffness values. Hence, a lower rate of stiffness reduction was observed for the IWPET modified asphalt mixes when the testing temperature increased relative to the RWPET modified asphalt mixes.

#### 3.2.2. Rutting of RWPET and IWPET Modified Asphalt Mixtures

**Figure 7:**
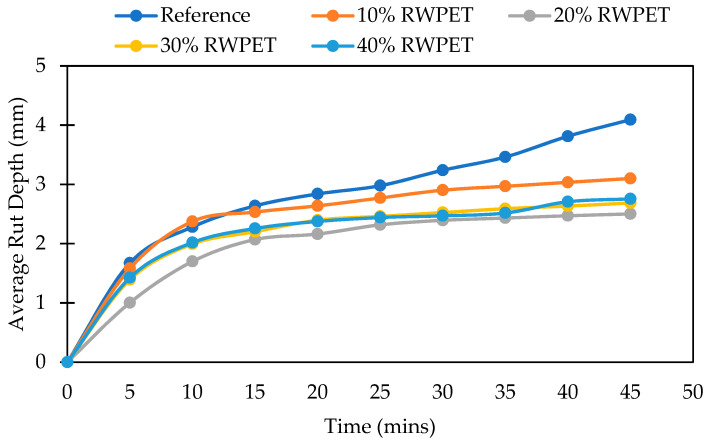
Average rut depth for RWPET modified mixtures at 45 °C.

**Figure 8:**
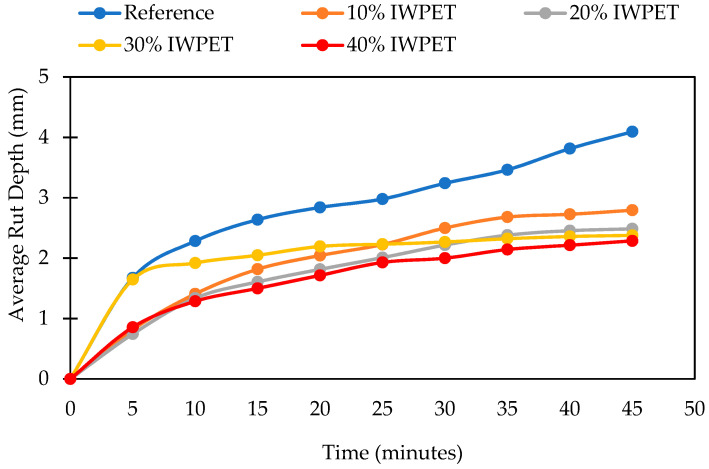
Average rut depth for IWPET modified mixtures at 45 °C.

The rutting depths of the nine different mixes considered in this study are presented in Figure 7 and Figure 8. The asphalt concrete mixtures containing both WPET granules (RWPET and IWPET) showed reduced rutting depth relative to the reference mixture, suggesting that the resistance to rutting distress of the modified asphalt mixtures was higher than that of the reference mixture. The increase in the rutting resistance of the modified asphalt mixes might be attributed to the nature of the WPET used, which was semi-crystalline. The WPET melt/softened during mixing due to the mixing temperature influence at its glass transition temperature and then mixed with the hot asphalt binder, which in turn improved its cohesion; likewise, the crystalline portion which remained preserve during mixing due to the elevated melting point of WPET served as elastic aggregates.

From Figure 7, it can be seen that the maximum enhancement in the rutting resistance of the RWPET modified asphalt mixes was attained at 20% RWPET content as substitutes for natural aggregates tested at 45 °C. However, as shown in Figure 8, for asphalt mixes containing IWPET granules, the maximum improvement in the rutting damage was obtained for mixtures manufactured with 40% IWPET granules. This increase in the magnitude of resistance to rutting of the IWPET modified asphalt mixtures compared to the RWPET modified asphalt mixtures could probably be due to the impact of treating the WPET with gamma-irradiation, which turned the WPET to be rigid and stiffer relative to the RWPET.

Additionally, incorporating 20% RWPET to the substitutes’ natural aggregates yielded the optimum increase in the tolerance of the modified asphalt mixture to rutting by 38.85%. However, when IWPET granules were incorporated as a substitute for natural aggregates, the modified mixture prepared with 40% IWPET granules showed the minimum rutting depth among the other modified asphalt mixes and the reference mixture, thus, suggesting that compared to other mixtures, the mixture containing 40% IWPET granules had better resistance to rutting distress. For modified mixtures containing 40% IWPET granules, the resistance to rutting depth was observed to be boosted by 49.6% compared to the reference mix.

The finding further demonstrates that higher amounts of IWPET granules had a substantial impact on the modified mixes’ elastic recovery by adding more elasticity that directly improved the recovery properties of mixtures under repeated loading [60]. This trend could be attributed to the elastic contact within the asphalt concrete mixes because of the addition of IWPET granules into the modified blends as natural aggregates substitutes that serve as elastic aggregates.

As shown in the XRD plot of waste PET after exposure to gamma-irradiation, the chain scission and crosslinking in WPET because of gamma-irradiation altered the surface characteristics of the material, which in turn produced superior strength properties, resulting in a stiffer mixture, which in turn increased the resistance of asphalt mixes to permanent deformation.

Based on the outcome from the stiffness and rutting of the asphalt mixture prepared with RWPET and IWPET granules as sustainable aggregate, it was concluded that treating the WPET through exposure to gamma ionizing radiation led to an enhancement in the performance and durability of the asphalt mixtures made with IWPET compared to the asphalt mixture made with RWPET. Thus, in the subsequent section, statistical analysis employing an RSM-ANN framework was conducted to model and predict the stiffness and rutting properties of the IWPET modified asphalt mixes considering test temperature and waste material content as input factors.

### 3.3. Statistical Modeling Using RSM

#### 3.3.1. Stiffness Modulus (S_m_) Response

Asphalt mixture stiffness is a critical variable of flexible pavement construction. It has been discovered that stiffness and other mixture characteristics, such as rutting and fatigue, are associated and can be considered a measure for assessing the asphalt concrete mixture efficiency. The relationship between asphalt mixture stress and strain has been demonstrated by the indirect tensile stiffness modulus (ITSM) of asphalt mixes, which can be utilized to assess the stiffness of asphalt mixture under complex environmental conditions. A fitted quadratic polynomial model to predict IWPET-modified asphalt mixture stiffness was generated after regression analysis was applied. The model was chosen depending on the polynomial of the highest order in which additional terms are relevant and not aliased by the software. The ANOVA analysis for the response surface quadratic model for stiffness is shown in Table 5.

The influences of input factors on the stiffness modulus of the IWPET-modified asphalt concrete mixture are displayed in Table 5. As shown in Table 5, the stiffness modulus response (*S_m_*) of the IWPET-modified mixture had a higher F-value of 129.60 and a significant *p*-value of less than 0.0001. The *p*-values of less than 0.05 for the model suggested that there was only a 0.01% probability that an F-value with this magnitude would occur resulting from noise. The 95% confidence interval was adopted to assess the importance of the model. All models were chosen according to the highest level in which the additional terms were essential and not aliased by the RSM software. The model terms $X_{1}$, $X_{2}$, $X_{1}X_{2}$, and $X_{1}^{2}$ had Prob > F values of less than 0.05, which means that the terms were essential for the modified asphalt mixture’s stiffness modulus, whereas the other term $X_{2}^{2}$ was insignificant as its *p*-value was more than 0.05. The insignificant *p*-value implied that the model term $X_{2}^{2}$ did not significantly affect the stiffness modulus of the modified asphalt concrete mixtures.

Moreover, the lack of fit (LoF) F-test was again employed to determine how adequate the model is. LoF represents the variance of the data on the model. For the model to be statistically fit, the *p*-value for the lack of fit must be greater than 0.05. For this response, the LoF was noted to have a *p*-value of 0.0347. It should be observed that, even though the LoF for the stiffness modulus (*S_m_*) was significant, a reasonable agreement was seen among the predicted and adjusted coefficient of determination, and, thus, the suggested model could be used to navigate the design space to find an optimum condition [31]. The final model equation for the stiffness modulus of the IWPET-modified asphalt concrete mixture is illustrated in Equation (5).

(5) $$S_{m}=+1806.28-1047.77X_{1}+123.92X_{2}-211.11X_{1}X_{2}-271.18X_{1}^{2}-115.06X_{2}^{2}$$

A model reduction through the backward elimination regression method was performed to remove the insignificant term, enhancing the model performance. The final equation for the stiffness modulus in terms of significant factors is shown in Equation (6).

(6) $$S_{m}=+1743.23-1041.86X_{1}+137.35X_{2}-190.74X_{1}X_{2}-273.55X_{1}^{2}\;$$

#### 3.3.2. Rutting (R_d_) Response

Rutting is another problematic distress caused by the accumulation of permanent deformation over time of one or more of the pavement layers under repeated traffic loading action in the flexible pavement at higher pavement temperature. Rutting in asphaltic pavements results in a significant decrease in the pavement’s structural and functional performance. In assessing their potential for rutting, the properties of pavement materials are important factors. In pavements, the rutting depth depends mostly on the stiffness of the asphalt concrete mixture. Table 5 presents the ANOVA analysis for the response surface quadratic model for rutting.

Table 5 presents the impacts of individual input variables on the rutting response (*R_d_*). The outcome from ANOVA showed that the rutting quadratic regression model was highly significant because its F-value of 105.16 was high and had a relatively low probability value (*p* < 0.0001), implying that the chosen model is significant statistically. This *p*-value means that a model F value of this magnitude could only be 0.01 percent likely to occur because of noise [32,61]. All the model terms $X_{1}$, $X_{2}$, $X_{1}X_{2}$, $X_{1}^{2}$, and $X_{2}^{2}$ were significant as their *p*-values were less than 0.05. These significant *p*-values showed that for the rutting response, all the terms had significant influence. The probability value of the LoF was reported as <0.00001, indicating that the LoF was significant. The established statistical model is shown in Equation (7) in terms of the coded variables for the IWPET-modified asphalt concrete mixture’s rutting. Moreover, it could be elucidated that the test temperature (*X*_1_) with the highest F-value of 198.96 compared to the IWPET granules (*X*_2_) with an F-value of 120.09 had the most significant influence on the rutting response.

(7) $$R_{d}=+2.13+0.9797X_{1}-0.7746X_{2}-0.3381X_{1}X_{2}+0.4589X_{1}^{2}+0.9030X_{2}^{2}.\;$$

#### 3.3.3. RSM ANOVA Model Validation

As shown in Table 6, the stiffness modulus response model had a very high determination coefficient R^2^ of 0.9789, which is very close to 1, showing the established model’s adequacy and quality. A higher value of R^2^ suggested a more robust agreement between the expected and the actual values. The predicted coefficients of determination (Pred. R^2^) were in acceptable agreement with their corresponding adjusted coefficients of determination (adj. R^2^) corresponding to less than 0.2 discrepancies. The lower value of the generated model’s standard deviation relative to its mean indicated its variance concerning the data from the test. For the model generated, therefore, experimental data produced less ambiguity. The signal-to-noise ratio of the model was estimated based on adequate precision (AP). A ratio greater than 4 is desirable and signifies that all the produced models can be used to navigate the design space [62]. The AP value for the stiffness modulus model was observed in this analysis to be 31.1336, showing that the model is reasonable, sufficient, and could be applied to navigate the design space.

The predicted R^2^ value of 0.9543, as shown in Table 6, was in realistic agreement with the adjusted R^2^ value of 0.9648, which implied a discrepancy of less than 0.2 between them. This lower difference between the coefficients of determination demonstrated that the model produced was sufficient. The signal to the various environmental factors (noise) ratio to which the laboratory tests were exposed estimated adequate precision (AP). A ratio greater than four for the model is ideal. The model formed was desirable and acceptable with the AP value of 33.4799. Depending on the discussion above, it could be reported that the established model was suitable for predicting the rutting response of the IWPET-modified asphalt mixture.

#### 3.3.4. Predicted vs. Actual Plots for the Responses

**Figure 9:**
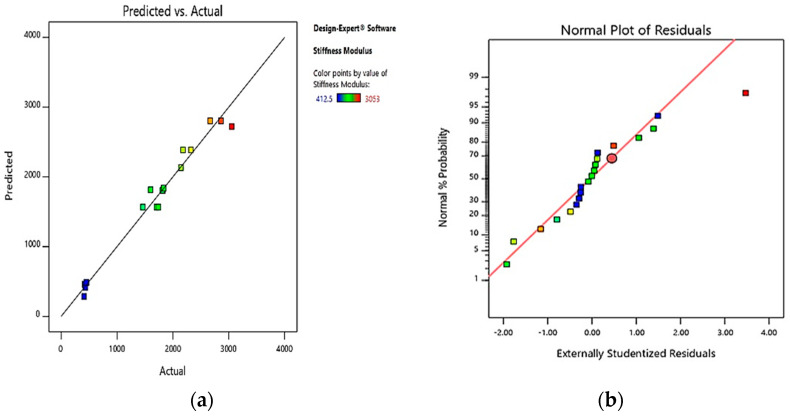
Stiffness modulus response plots for (**a**) predicted against actual, (**b**) normal plot of residuals.

For the developed stiffness modulus model, the predicted versus actual plot is shown in Figure 9a and was utilized to verify the fitness and degree of accuracy. The data points were, as shown in Figure 9a, scattered relatively near the equality line. The spread of data points along the straight line indicated the models’ acceptable fitting accuracy, and the actual and expected outcomes agreed with one another. Additionally, the output model established was accurate and acceptable in calculating the IWPET-modified asphalt concrete mixture’s stiffness modulus. Figure 9b displays the plot of normal percentage for the stiffness modulus model versus externally studentized residuals. It can also be noted that the normal plot of residuals was adequate since most of the response points were entirely on the quality line.

**Figure 10:**
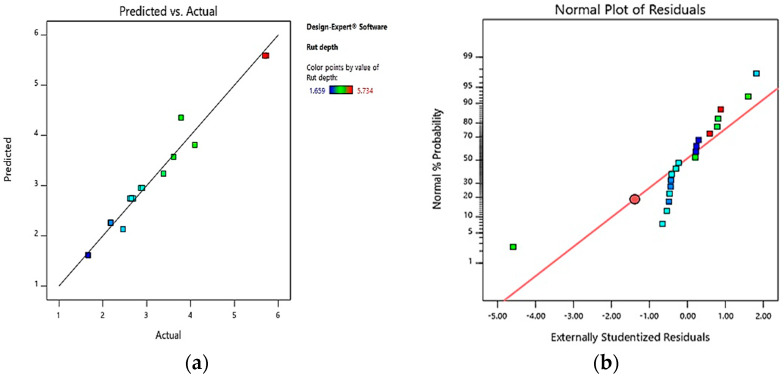
Rutting response plots for (**a**) predicted against actual, (**b**) normal plots of residuals.

The correlation between the rutting predicted and actual laboratory findings are displayed in Figure 10a. The uniform spreading of the data points around the 45-degree line indicated that the model predicted the rutting response accurately as the predicted model data were well-matched with the actual laboratory data. In order to assess the spread of the outcomes, Figure 10b was plotted. As depicted in Figure 10b, the data were relatively lying within the equality line and were remarkably symbolized by the S-shaped distribution. In the error definition, normality was indicated by a linear pattern, and no signs of faults were displayed by the experimental results, which verified that the outcomes were distributed normally.

### 3.4. Effects of Interactive Variables

#### 3.4.1. Stiffness Modulus Response

**Figure 11:**
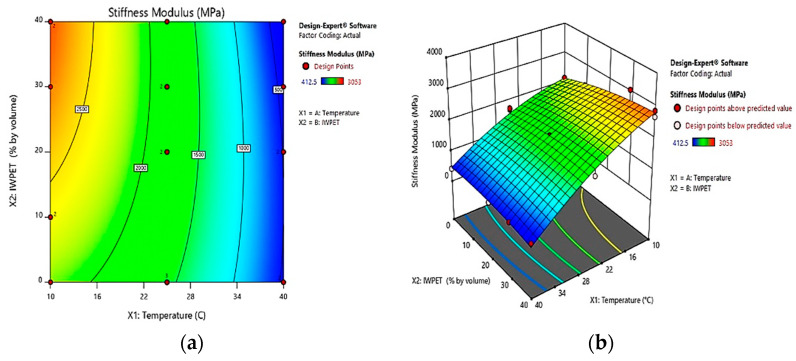
Influence of temperature and IWPET on stiffness (**a**) 2-dimensional, (**b**) 3-dimensional.

The 2-dimensional contour plots for the stiffness modulus model of the IWPET-modified asphalt concrete mixtures are shown in Figure 11a. The overall contour lines were found to be semi-elliptical in shape, suggesting a strong relationship between the temperature of the test and IWPET. The model was observed to show an excellent synergetic impact between the input parameters. The contour plots showed that the stiffness modulus decreased rapidly as the test temperature rose and increased slightly with the IWPET amounts. Additionally, an optimum output area with the highest stiffness modulus value was shown by the yellowish-red section on the 2-D plot containing IWPET particles. In contrast, the deep blue portion in the contour plot represented the area with the least impact on the response parameter value, in which case the optimum output region was approximately 20–40% IWPET and 10–16 °C test temperature, where the stiffness modulus value was higher with varying amounts of the input factors.

The effects of the two independent variables considered in this study (i.e., testing temperature and IWPET content) on the mixture’s stiffness modulus are illustrated in Figure 11b. From the figure, it can be seen that the mixture stiffness value was more sensitive to changes in temperature than the IWPET amounts. The stiffness modulus values decreased significantly when the testing temperature was increased from 10 °C to 40 °C. Moreover, the IWPET contents showed more influence on the mixture stiffness at lower temperatures. This behavior could be due to the asphalt binder’s sensitivity to temperature change, which had a vital function in the mixture’s properties. In other words, the asphalt binder got soft as the environmental temperature rose, which could ultimately lead to reduced stiffness of the mixture. Further, the IWPET granules’ influence on the stiffness of the mixture was superseded by the temperature.

#### 3.4.2. Rutting Response

**Figure 12:**
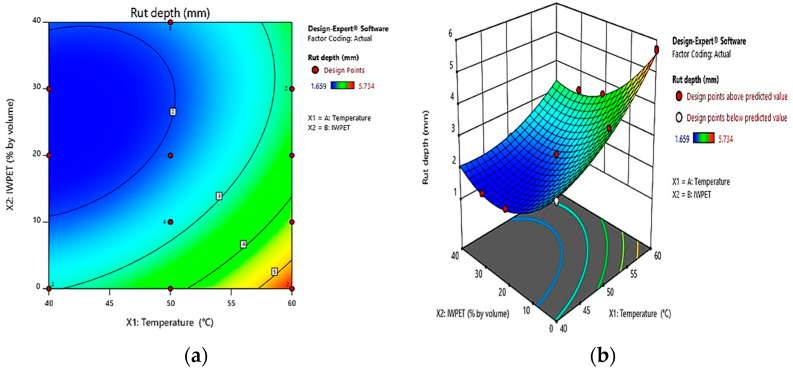
Influence of temperature and IWPET on rutting (**a**) 2-dimensional, (**b**) 3-dimensional.

The 2-dimensional contour diagrams were also used to assess the relationships between the inputs and response variables. Figure 12a presents the 2-dimensional contour diagram for rutting response. It can be demonstrated that the contour response plot also showed an oval shape signifying a good interaction between the independent factors. From the contour diagram, it can be noted that the rutting substantially reduced with increasing the IWPET content. The rutting decreased markedly by increasing IWPET from 10% to 40%, and it increased significantly when the test temperature was increased from 40 °C to 60 °C. Furthermore, the optimum performance region could be seen around 40–45 °C test temperature and 30–40% IWPET content by volume of equal size, where rutting response was lowest.

Figure 12b illustrates the 3-dimensional response surface plot for rutting response with the correlative impact of the two autonomous parameters, environmental factor (test temperature) and IWPET content. It can be noticed that, compared to the mixture prepared without IWPET, the reduction in rutting depth when IWPET particles were added at ranges between 30 and 40% was relatively high, suggesting that IWPET had a relatively significant influence on the rutting of the modified asphalt mixture. However, the environmental condition remarkably affected the rutting as the rut depth increased linearly with increased test temperature from 40 °C to 60 °C. Additionally, it can be seen from the 3-D contour graph that the lowest rut depth was obtained with the mixtures made with the highest IWPET percentage by volume and lower test temperature. The elastic properties of the IWPET granules under compaction loading could be attributed to this pattern as well.

### 3.5. Optimization and Validation of the RSM-Developed Models

#### 3.5.1. Stiffness Modulus

**Figure 13:**
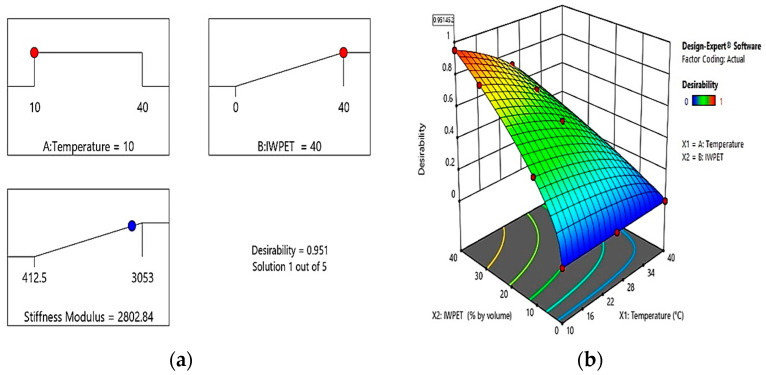
(**a**) Optimization ramps for the stiffness response; (**b**) desirability plot.

In this investigation, numerical optimization was performed to determine the optimum independent variables contents and the proposed models’ accuracy. The desired goals and ranges of the optimization are presented in Table 7, while the independent variables’ optimum values and the maximum predicted responses are presented in Figure 13a. The optimal test temperature and IWPET granules were predicted to be 10 °C and 40%, respectively, with the desirability of 0.951. The predicted model accuracy was validated by conducting an extra set of experiments with three replicate specimens at the globalized optimum independent parameters obtained via the numerical optimization analysis and comparing the predicted and actual responses. The optimum solution combinations were chosen based on a 1.0 overall desirability value. Figure 13a illustrates the optimization ramps for test temperature-IWPET granules contents and desirability value. The optimization ramp revealed the desirability of dependent variables ranging from 0 to 1. Every dot on the ramp shows the desired target for variable and response behaviors. Additionally, Figure 13b displays the 3-dimensional graph for the results of numerical optimization.

The validation test results after optimization revealed that the predicted stiffness modulus of the IWPET-modified asphalt mixture was found to be 2802.84 MPa, and the actual stiffness modulus at the globalized optimum input parameters was observed to be 2861 MPa. The absolute relative percentage error (*APE*) between the actual and optimally predicted value was measured utilizing Equation (8) as a predictability measure and found to be 2.03%. As can be seen, the absolute percent error between the actual and predicted value of the stiffness modulus response was observed to be very small, less than 2.1%. As a result, the model predicted the response with strong accuracy.

(8) $$APE=\left|1-\frac{Predicted\;model}{Actual\;model}\right|\times 100\%$$

#### 3.5.2. Rutting

**Figure 14:**
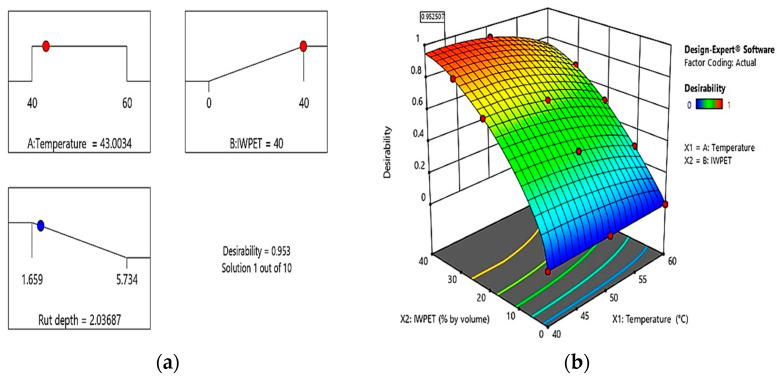
(**a**) Optimization ramps for the rutting response; (**b**) desirability plot.

After the ANOVA analysis and validation, the multi-objective optimization approach comprising RSM was used as the basis for determining the best solution in this analysis. The primary objective of optimization was to identify the appropriate mixture of independent parameters that could be utilized to achieve an optimized IWPET-modified asphalt mixture based on the maximum rutting performance. All parameters were established within the stated interval to capture all feasible solutions and optimize the outputs. Table 8 presents the optimization thresholds for the input parameters and the responses. In Figure 14a, the numerical optimization ramp for the response rutting is shown. The optimized rutting of the modified asphalt mixture could be obtained by utilizing 43 °C as the testing temperature and incorporating 40% by volume IWPET granules as a substitute for conventional aggregate. The optimized rutting depth value was 2.037 mm. As shown in Figure 14b, the optimized solution had high desirability of 95.3%.

To verify the suitability of the optimization results obtained by the software Design-Expert and the whole response model, an additional series of laboratory tests were performed by utilizing the optimized levels of input factors and two more distinct mixes to verify the optimized mixture proportion of the design mixes. Consequently, three samples were prepared for the test parameter, and their average value was measured and recorded. The rutting depth response was tested, and a comparison was made with the predicted results. The absolute percentage error (*APE*) between the experimental and predicted results was calculated using Equation (8) above. The actual rutting was determined using the optimized test temperature and IWPET as 2.077 mm.

The actual result was in good agreement with the predicted result with an average absolute percentage error of less than 2% based on the calculated percentage error.

### 3.6. Artificial Neural Network Model

**Figure 15:**
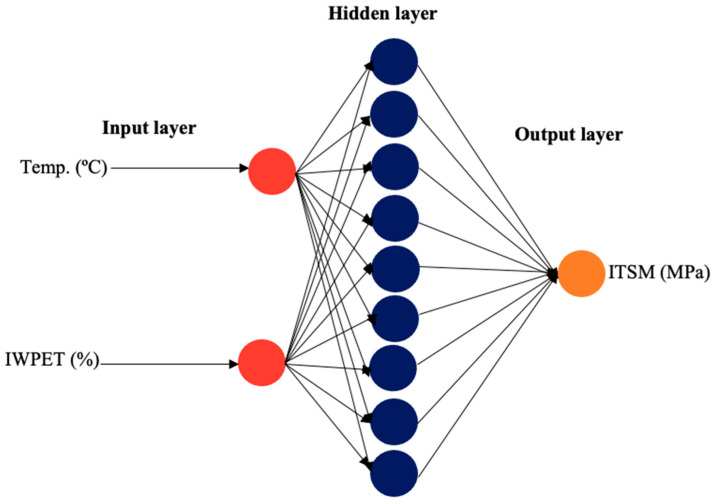
ANN topology for the stiffness modulus response of the modified asphalt mix.

**Figure 16:**
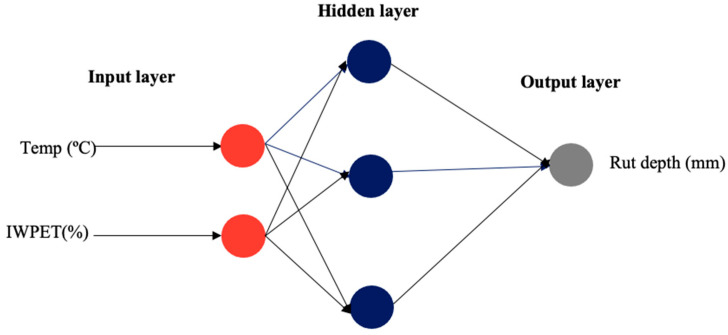
ANN topology for the rutting response of the modified asphalt mix.

The ANN architectural model application for the input combination has been an essential tool for accurately determining the data set with minimal or negligible error [63]. In this study, the neural network was employed for estimating the performance properties of IWPET-modified asphalt concrete mixtures. Environmental condition (temperature) and IWPET granules content by volume of equal size were selected as input factors, while the output factors stiffness modulus (*S_m_*) and rutting (*R_d_*) were selected separately because of the different range of testing conditions. Logsig transfer function, Levenberg–Marquardt (Trainlm) training function, and the gradient descent with momentum weight and bias learning function (LEARNGDM) were used for creating the ANN. The feed-forward backpropagation network type was applied, which is generally utilized to describe complicated system modeling and identification [64]. Thus, the number of neurons was varied accordingly to obtain the best network architecture of the ANN hidden layer. For both responses considered in this work, a three structural layer with 2-9-1 and 2-3-1 designed topology, as displayed in Figure 15 and Figure 16, was found to be the best based on the statistically highest R^2^ values (0.9997 and 0.9995) and least RMSE values (7.887 and 0.0172) for the stiffness modulus (*S_m_*) and rutting (*R_d_*), respectively, as illustrated in Table 9.

The respective statistical values of other architectural neurons employed are presented in Table 9. The input neurons comprised the two studied parameters: test temperature and IWPET content. The second layer (hidden) represented the ninth and third hidden neurons, while the third layer (output) was the predicted response for the performance properties of the modified asphalt concrete mixture, including stiffness modulus (*S_m_*) and rutting (*R_d_*), respectively.

**Figure 17:**
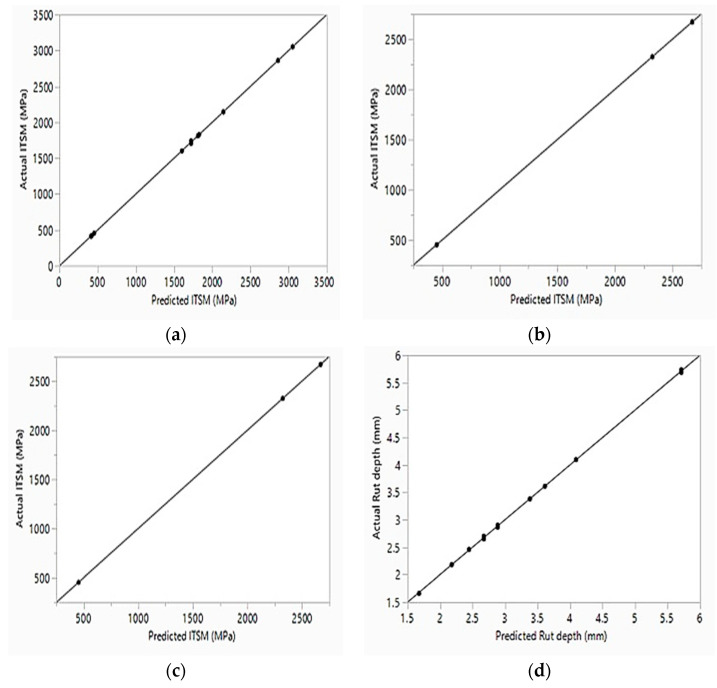

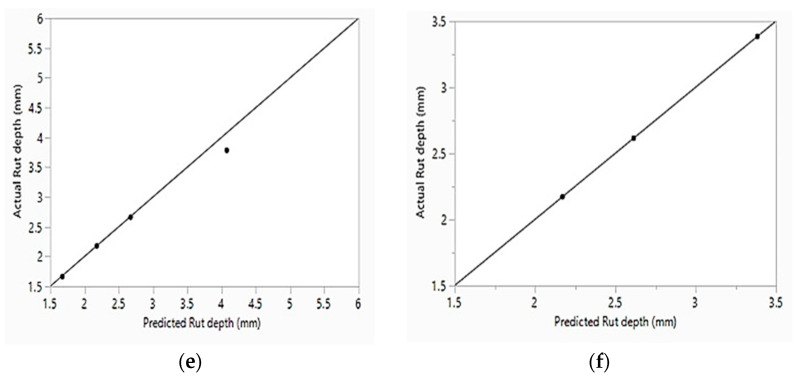
ANN model actual vs. predicted for the response stiffness modulus: (**a**) training, (**b**) validation, (**c**) testing and the response rutting depth, (**d**) training, (**e**) validation, (**f**) testing.

The graph for all input and output variables for the stiffness modulus (*S_m_*) that compares the test data against the ANN data in training, validation, and testing the predictions networks are shown in Figure 17a–c with very high values of R^2^ (0.9997, 0.9500, and 0.9180, respectively). Similarly, the scatter plots for the rutting (*R_d_*) response are presented in Figure 17d–f. This figure shows that the determination coefficients for the training, validation, and testing were 0.9995, 0.9660, and 0.9970, respectively. According to these values, ANN gave high accuracy results in modeling the output parameters. Nearly all data were spread over the 45° line that is a sign of perfect suitability among the test results and the ANN predicted results. The values of the R^2^ among test and ANN-predicted outputs pointed out clearly that the applied ANN model trained with the test data was accurate in estimating the stiffness modulus and rutting depth of the modified asphalt concrete mixes.

### 3.7. RSM and ANN Models Validation and Comparison

Design of Experiment (DoE) depending on response surface methodology (RSM) and artificial neural network (ANN) has been the most adopted modeling and process optimization techniques in recent years [65]. In this research, RSM and ANN approaches were used to predict two different IWPET-modified asphalt concrete mixture responses, considering two input factors. The associations between the actual and predicted values were verified to authenticate the appropriateness of the statistical models. Based on the analysis performed in the previous section utilizing both RSM and ANN to model the stiffness modulus and rutting depth of the IWPET-modified asphalt concrete mixture, it was noted that both statistical models were adequate and satisfactory to reflect the expected outcomes. The actual and predicted outcomes and their corresponding absolute percentage errors (*APE*) using both RSM and ANN models are shown in Table 10.

The performance index parameters values of the model are displayed in Table 11. It can be shown that the correlation coefficient (R^2^) of the ANN prediction model was higher than that obtained by the RSM model, and the error prediction values of the ANN model were observed to be minimal than the RSM model for both responses considered. Therefore, comparing the evaluated performance index parameters presented in Table 11 of both models, it can be concluded that the ANN model gave more accurate outputs than the RSM model for data fitting and estimation capabilities of the IWPET-modified asphalt concrete mixtures stiffness modulus and rutting characteristics. However, the latter can still be used to optimize the performance properties of the modified asphalt mixtures. The ability of the ANN model to imitate human intelligence in learning the data set pattern was attributed to its superior performance over the RSM model [66].

## 4. Conclusions

In this current work, the feasibility of incorporating RWPET and IWPET granules as sustainable aggregate in densely graded asphalt mixture, was evaluated and the impact of environmental condition (temperature) and the replacement percentage by volume of IWPET granules on the stiffness and rutting characteristics of IWPET-modified asphalt blends were investigated by RSM and ANN. The following are the most relevant conclusions made from this investigation:

• The XRD and DSC analysis justified the increase in crystallinity degree and thermal stability phenomena of WPET due to exposure to gamma-irradiation. After exposure to 100 kGy gamma-irradiation dosage, the crystallinity degree of the WPET increased from 41.15 to 46.07% due to the effect of chain scission. On the other hand, the same transmittance bands were identified for both materials through the FT–IR analysis, indicating that the irradiation did not alter the peak positions and chemical bonds of the WPET.
• The obtained results from the stiffness and rutting characteristics indicate that the stiffness and rutting resistance were boosted for both WPET modified asphalt mixtures. Comparing the RWPET modified asphalt mixes with IWPET modified asphalt mixes, it was noted that IWPET modified asphalt mixes showed better and improved stiffness and rutting resistance. Additionally, With the gamma-irradiation process, the utilization of WPET could be increased from 20% to 40% (equivalent to 5% to 10% by weight of the mix), which could save the natural resources from depleting and landfilling space with significant enhancement in the stiffness and rutting resistance of the modified mixtures.
• Both independent factors influenced the stiffness modulus and rutting properties. However, mixture stiffness was more prone to changes in temperature than the IWPET amount. The rutting was enhanced as the IWPET content was increased at all test temperatures, implying that incremental increases in the amount of IWPET percentage by volume could increase the resistance to permanent deformation of the asphalt mixtures.
• Based on experimental findings, the application of RSM and ANN models showed that they were reliable and practical models for predicting the stiffness modulus and rutting of asphalt concrete mixtures containing IWPET alternative aggregates. Comparing the two statistical models indicated that the ANN model is better than the RSM model for the two responses considered, having a higher determination coefficient (R^2^) and minimum prediction errors (RMSE and MRE) than that achieved via the RSM.
• With respect to treating the WPET granules with gamma-irradiation, more waste could be incorporated as conventional materials supplement in asphalt concrete mixes without compromising the mechanical performance properties. The incorporation of an increased volume of WPET for green and cleaner pavement as a result of gamma-irradiation treatment could be an effective approach to recycling instead of traditional methods of recycling. Recycling and gamma-irradiation treatment of WPET in asphalt mixtures will contribute to a cleaner and sustainable pavement.

For future investigations, it is recommended to perform other mechanical performance tests such as fatigue cracking, indirect tensile strength (ITS), and semi-circular bending (SCB) for the regular and gamma-irradiated waste polyethylene terephthalate modified asphalt mixtures.

## Figures and Tables

**Table 1:** Physicochemical properties of aggregate and asphalt binder.

Property	Method	Result	Limits
Coarse aggregate			
L.A. Abrasion (%)	ASTM C131	22.5	<30
Elongation index (%)	BS 812-105.2	13.23	<20
Flakiness index (%)	BS EN 933-3	7.4	<20
AIV (%) ^1^	BS 812-3	11.2	<15
ACV (%) ^2^	BS 812-110	19.81	<20
Specific gravity	ASTM C128	2.66	-
Water absorption (%)	ASTM C128	0.50	<2.0
Fine aggregate			
Specific gravity	ASTM C128	2.63	-
Water absorption (%)	ASTM C128	1.23	<2.0
Asphalt binder			
Penetration at 25 °C (0.1 mm)	ASTM D5-13	64	60–70
Softening point (°C)	ASTM D36-12	49	49–56
Ductility at 25 °C (cm)	ASTM D113	120	Min 100
Density (g/cm^3^)	ASTM D70	1.02	1.00–1.06
Mass loss (%)	ASTM D2872	0.12	<1.00

^1^ Aggregate impact value; ^2^ aggregate crushing value.

**Table 2:** WPET gradation utilized.

Size of Sieve (mm)	% Passing
3.35	100
2.36	89
1.18	0

**Table 3:** Marshall Mix design results for the matrix tested.

Mix Type	AC (%)	BSD	AV (%)	VMA (%)	VFA (%)	MS (kN)	MF (mm)	OAC (%)
Reference	4.0	2.3273	7.2419	16.15	55.1585	12.62	2.86	5.27
	4.5	2.3823	4.3867	14.62	69.9911	14.34	2.94	
	5.0	2.3947	3.2288	14.62	77.9197	14.78	3.05	
	5.5	2.4005	2.3393	14.87	84.2683	15.28	3.11	
	6.0	2.3997	1.7241	15.34	88.7608	13.76	3.26	
	6.5	2.3900	1.4799	16.14	90.8309	10.59	3.38	
10% RWPET	4.0	2.3193	8.1869	16.18	49.4011	12.11	3.04	5.07
	4.5	2.3697	5.0256	15.68	67.9490	13.16	3.12	
	5.0	2.3851	4.3031	15.18	71.65283	13.84	3.19	
	5.5	2.3748	2.7327	14.98	81.7577	13.33	3.21	
	6.0	2.3722	2.5420	16.31	84.4145	12.71	3.29	
	6.5	2.3698	1.8921	16.85	88.7709	11.69	3.45	
20% RWPET	4.0	2.2835	8.6082	17.74	51.4758	11.75	3.12	4.98
	4.5	2.3306	5.1538	15.87	67.5249	12.77	3.18	
	5.0	2.3451	4.5640	15.99	71.4572	13.09	3.29	
	5.5	2.3373	3.0570	16.33	81.2799	12.26	3.36	
	6.0	2.3299	2.7820	16.84	83.4798	11.81	3.46	
	6.5	2.3186	2.0189	17.8	88.6579	10.89	3.55	
30% RWPET	4.0	2.2415	9.1000	18.27	50.1916	11.07	3.14	4.93
	4.5	2.2856	5.3700	17.10	68.5965	11.55	3.22	
	5.0	2.3101	5.0400	16.65	69.7297	12.05	3.29	
	5.5	2.2993	3.1800	17.48	81.8078	11.77	3.39	
	6.0	2.2819	2.7900	18.53	84.9433	10.97	3.48	
	6.5	2.2674	2.0420	19.48	89.5175	9.79	3.57	
40% RWPET	4.0	2.1995	9.3500	19.81	52.8016	10.30	3.15	4.90
	4.5	2.2482	5.3800	18.46	70.8559	10.86	3.25	
	5.0	2.2751	5.1100	17.91	71.4684	11.19	3.31	
	5.5	2.2603	3.1600	18.88	83.2627	10.75	3.43	
	6.0	2.234	2.8000	20.24	86.1660	9.97	3.52	
	6.5	2.2158	2.0850	21.32	90.2205	9.58	3.60	
10% IWPET	4.0	2.2880	7.7568	16.58	53.2125	14.47	3.02	5.17
	4.5	2.3657	3.9622	14.20	72.0878	16.22	3.10	
	5.0	2.3687	3.1840	14.54	78.0958	15.97	3.21	
	5.5	2.3700	2.4812	14.94	83.3915	15.54	3.27	
	6.0	2.3695	1.8597	15.41	87.9298	13.97	3.44	
	6.5	2.3690	1.2423	15.88	92.1745	11.44	3.59	
20% IWPET	4.0	2.2555	7.9989	17.76	54.9709	15.58	3.13	5.12
	4.5	2.3196	4.7353	15.87	70.1565	16.99	3.21	
	5.0	2.3285	3.7213	15.99	76.7222	16.02	3.32	
	5.5	2.3313	2.9636	16.33	81.8499	15.60	3.38	
	6.0	2.3293	2.4132	16.84	85.6720	13.99	3.55	
	6.5	2.3148	2.3950	17.80	86.5446	12.20	3.66	
30% IWPET	4.0	2.2290	8.0028	18.73	57.2727	16.29	3.44	5.07
	4.5	2.2773	5.3688	17.40	69.1473	17.70	3.52	
	5.0	2.2995	3.8027	17.03	77.6743	17.53	3.63	
	5.5	2.3034	3.0066	17.33	82.6506	16.31	3.69	
	6.0	2.3008	2.4837	17.86	86.0934	14.70	3.86	
	6.5	2.2879	2.4142	18.75	87.1278	12.89	3.97	
40% IWPET	4.0	2.1985	8.1701	19.84	58.8243	14.20	3.69	5.01
	4.5	2.2476	5.4836	18.48	70.3246	14.99	3.79	
	5.0	2.2686	3.9665	18.15	78.1434	14.64	3.86	
	5.5	2.2720	3.1914	18.46	82.7083	13.75	3.97	
	6.0	2.2734	2.5087	18.84	86.6828	13.61	4.08	
	6.5	2.2612	2.4167	19.70	87.7343	12.12	4.24	

**Table 4:** Matrix of experimental design for stiffness and rutting responses.

Run	Input Variables		Stiffness Modulus (MPa)	Input Variables		Rutting (mm)
	X1: Temp. (°C)	X2: IWPET (%) by Vol.		X1: Temp. (°C)	X2: IWPET (%) by Vol.	
1	40	40	412.50	40	20	1.668
2	25	30	1839.0	50	40	2.171
3	40	30	432.00	60	30	3.384
4	10	30	3053.0	50	10	2.614
5	25	30	1828.0	50	0	4.095
6	25	0	1463.0	40	30	1.662
7	40	20	453.20	40	30	1.659
8	25	20	1817.0	60	10	3.784
9	10	0	2145.75	60	20	3.612
10	10	40	2861.0	60	30	3.380
11	40	0	417.50	50	10	2.662
12	25	0	1704.0	50	10	2.651
13	25	40	1602.5	40	0	2.863
14	25	0	1742.0	50	40	2.179
15	40	0	424.5	60	0	5.734
16	25	20	1813.0	50	40	2.181
17	10	40	2670.0	50	20	2.458
18	10	10	2179.5	40	0	2.902
19	10	10	2324.0	60	0	5.687
20	40	20	453.9	50	10	2.699

**Table 5:** ANOVA results for responses.

Source	Stiffness Modulus (MPa)					Rutting (mm)				
	Sum of Squares	Df	Mean Square	F-Value	*p*-Value	Sum of Squares	Df	Mean Square	F-Value	*p*-Value
Model	1.413 × 10^7^	5	2.825 × 10^6^	129.60	<0.0001	24.71	5	4.94	105.16	<0.0001
*X*_1_	1.296 × 10^7^	1	1.296 × 10^7^	594.35	<0.0001	9.35	1	9.35	198.96	<0.0001
*X*_2_	1.654 × 10^5^	1	1.654 × 10^5^	7.59	0.0155	5.64	1	5.64	120.09	<0.0001
*X*_1_ *X*_2_	2.889 × 10^5^	1	2.889 × 10^5^	13.25	0.0027	0.5340	1	0.5340	11.36	0.0046
*X*_1_^2^	3.490 × 10^5^	1	3.490 × 10^5^	16.01	0.0013	0.9976	1	0.9976	21.23	0.0004
*X*_2_^2^	44,874.92	1	44,874.92	2.06	0.1733	2.60	1	2.60	55.23	<0.0001
Residual	3.052 × 10^5^	14	21,799.87			0.6579	14	0.0470		
LoF	2.306 × 10^5^	6	38,439.26	4.12	0.0347	0.6523	5	0.1305	209.42	<0.0001

Where $X_{1}$: testing temperature, $X_{2}$: IWPET, $X_{1}X_{2}$: interaction effect, $X_{1}^{2}$, $X_{2}^{2}$: second-order effect, df: degree of freedom, F-values: Fisher-statistical test values, *p*-values: probability values, LoF: lack of fit.

**Table 6:** ANOVA results for responses.

Response	Std. Dev.	Mean	C.V.%	R^2^	Adjusted R^2^	Predicted R^2^	Adequate Precision
Stiffness Modulus (MPa)	147.65	1581.77	9.33	0.9789	0.9713	0.9530	31.1336
Rutting (mm)	0.2168	3.00	7.22	0.9741	0.9648	0.9543	33.4799

**Table 7:** Numerical optimization selected criteria for stiffness modulus response.

Input and Output Parameters	Unit	Desired Goals	Lower Band	Upper Band
Test temperature	°C	in range	10	40
IWPET	%	maximize	0	40
Stiffness Modulus	MPa	maximize	412.5	3053

**Table 8:** Optimization thresholds for rutting response.

Input and Output Parameters	Unit	Desired Goals	Lower Band	Upper Band
Test temperature	°C	in range	10	40
IWPET	%	maximize	0	40
Rutting	mm	maximize	1.659	5.734

**Table 9:** Optimized ANN architecture for input and output factors prediction.

No.	Nodes	Stiffness Modulus (MPa)		Nodes	Rutting Depth (mm)	
		R^2^	RMSE		R^2^	RMSE
1	[3]	0.997	45.014	[3]	0.999	0.0172
2	[4]	0.992	67.236	[4]	0.984	0.1293
3	[5]	0.999	30.848	[5]	0.999	0.0227
4	[6]	0.999	29.829	[6]	0.994	0.0911
5	[7]	0.995	57.039	[7]	0.998	0.0444
6	[8]	0.998	35.777	[8]	0.999	0.0276
7	[9]	0.999	7.887	[9]	0.984	0.1619
8	[10]	0.993	61.798	[10]	0.955	0.2807

**Table 10:** Comparison between ANN and RSM prediction for the responses.

Stiffness Modulus (MPa)					Rutting Depth (mm)				
Actual	ANN		RSM			ANN		RSM	
	* Pred.	** APE	Pred.	APE	Actual	Pred.	APE	Pred.	APE
412.50	412.50	0.00	412.50	0.00	1.668	1.751	4.74	1.659	0.54
1839.0	1828.0	0.60	1839.50	0.03	2.171	2.1711	0.005	2.261	3.98
432.00	445.40	3.01	414.97	3.94	3.384	3.385	0.03	3.241	4.23
3053.0	3052.1	0.03	2871.62	5.94	2.614	2.614	0.00	2.746	4.81
1828.0	1828.2	0.01	1839.47	0.62	4.095	4.091	0.10	3.810	6.96
1463.0	1463.0	0.00	1567.29	6.65	1.662	1.677	0.89	1.659	0.18
453.20	453.20	0.00	487.33	7.00	1.659	1.677	1.07	1.659	0.00
1817.0	1813.0	0.22	1806.28	0.59	3.784	4.074	7.12	4.153	8.89
2145.8	2145.74	0.001	2132.77	0.61	3.612	3.6122	0.01	3.571	1.14
2861.0	2861.05	0.002	2802.84	2.03	3.380	3.382	0.06	3.241	4.11
417.50	421.00	0.83	459.46	9.13	2.662	2.673	0.41	2.746	3.06
1704.0	1723.0	1.10	1567.29	8.02	2.651	2.673	0.82	2.746	3.46
1602.5	1602.45	0.003	1815.13	11.71	2.863	2.886	0.80	2.952	3.02
1742.0	1723.0	1.09	1567.29	10.03	2.179	2.180	0.05	2.261	3.63
424.5	421.00	0.82	459.46	7.61	5.734	5.712	0.38	5.587	2.56
1813.0	1813.11	0.006	1806.28	0.37	2.181	2.179	0.09	2.261	3.54
2670.0	2670.07	0.003	2802.84	4.74	2.458	2.446	0.49	2.313	5.90
2179.5	2324.0	6.22	2386.59	8.68	2.902	2.886	0.55	2.952	1.69
2324.0	2323.5	0.02	2386.59	2.62	5.687	5.712	0.44	5.587	1.76
453.9	453.95	0.01	487.33	6.86	2.699	2.673	0.96	2.746	1.71

* Predicted, ** absolute percentage error.

**Table 11:** Comparison of RSM and ANN statistical performance index parameters.

Statistical Parameters	Stiffness Modulus (MPa)		Rutting Depth (mm)	
	RSM	ANN	RSM	ANN
R^2^ (%)	97.89	99.97	97.41	99.95
RMSE (MPa)	102.968	33.124	0.138	0.069
MRE (%)	4.859	0.699	3.259	0.951

## Data Availability

All the data are available within this manuscript.
